# Quantifying mitochondrial DNA copy number using robust regression to interpret real time PCR results

**DOI:** 10.1186/s13104-017-2913-1

**Published:** 2017-11-13

**Authors:** Paulo Refinetti, David Warren, Stephan Morgenthaler, Per O. Ekstrøm

**Affiliations:** 1 Ecole Polytechnique Féderale de Lausanne, 1015 Lausanne, Switzerland; 2 Department of Medical Biochemistry, Radiumhospital, 0379 Oslo, Norway; 3 Department of Tumor Biology, Radiumhospital, 0379 Oslo, Norway

**Keywords:** rtPCR, Robust regression, Mitochondrial DNA

## Abstract

**Background:**

Real time PCR (rtPCR) is a quantitative assay to determine the relative DNA copy number in a sample versus a reference. The $\Delta C_T$ method is the standard for the analysis of the output data generated by an rtPCR experiment. We developed an alternative based on fitting a robust regression to the rtPCR signal. This new data analysis tool reduces potential biases and does not require all of the compared DNA fragments to have the same PCR efficiency.

**Results:**

Comparing the two methods when analysing 96 identical PCR preparations showed similar distributions of the estimated copy numbers. Estimating the efficiency with the $\Delta C_T$ method, however, required a dilution series, which is not necessary for the robust regression method. We used rtPCR to quantify mitochondrial DNA (mtDNA) copy numbers in three different tissues types: breast, colon and prostate. For each type, normal tissue and a tumor from the same three patients were analysed. This gives a total of six samples. The mitochondrial copy number is estimated to lie between 200 and 300 copies per cell. Similar results are obtained when using the robust regression or the $\Delta C_T$ method. Confidence ratios were slightly narrower for the robust regression. The new data analysis method has been implemented as an R package.

**Electronic supplementary material:**

The online version of this article (10.1186/s13104-017-2913-1) contains supplementary material, which is available to authorized users.

## Background

Mitochondria are the organelle responsible for most of the energy production in eukaryotic cells. Each mitochondrion carries several copies of mitochondrial DNA, which is composed of a single circular chromosome of 16569 base pairs (hg38, GRCh38, Dec. 2013). It encodes for 22 tRNA, 13 protein subunits and two ribosomal RNA subunits. There are currently few accurate measurements of mtDNA copy number in cells [1–5], even though this number affects the symptoms of mitochondrial diseases [6–9]. Better measurements of mtDNA copy numbers would improve the understanding of mtDNA mutagenesis [10–12] as well as the process through which mutations become homoplasmic. Mitochondrial mutations also appear to be involved in cancer development [13–17], and aging [18–21]. Furthermore, most tumors are thought to rely on glycolysis rather than oxidative phosphorylation for the majority of their energy, a process that could be related to mtDNA copy number. The standard method for quantifying DNA copy number is real time PCR (rtPCR) [22–24]. Most methods rely on amplifying a mitochondrial and a nuclear fragment in separate reactions, with the template from the same sample [16, 24]. Although there has been much development in the data analysis algorithms applied to rtPCR output, some challenges remainx [25, 26].

## Materials and methods

### Tissue and DNA extraction

Anonymous surgical discards were obtained after standardised informed consent. Tissue was stored at the surgical department at − 70 °C until DNA extraction. Normal and tumor tissue was obtained from three different patients with three different tumor types (breast, prostate and colon). The normal tissue was taken at a distance of 10–15 cm from the location of the tumor. A few milligrams were taken from each sample and had their DNA extracted.

### DNA extraction

Samples were digested with proteinase K for 4 h at 57 °C in 300 $\mu$l of digestion Buffer (Qiagen, Hilden, Germany) according to manufacturer’s instructions. DNA was extracted from them using the Qiagen MagAttract DNA Mini-M48 Kit with a dedicated automatic solution also provided by Qiagen. The result is a DNA solution containing approximately 50 ng of DNA per $\mu$l .

### Primers

**Table 1:** Primer sequences used to amplify nuclear and mitochondrial DNA sequences

Primer	Sequence (5′ to 3′)	Genome region
Mitochondrial forward	ACA CCC TCC TAG CCT TAC TAC	chrM: 10087–10192
Mitochondrial reverse	GAT ATA GGG TCG AAG CCG C	
Nuclear forward	AGG GTA TCT GGG CTC TGG	chr11: 2170993–2171170
Nuclear reverse	GGC TGA AAA GCT CCC GAT TAT	

Primers were designed using the rtPCR primer design tool of IDT (integrated DNA technologies). The nuclear and mitochondrial primer pairs were designed for simultaneous amplification. Table 1 shows the primer pairs. PCR conditions were optimised by testing various annealing temperatures, reaction volumes, and reagent concentrations. The objective was to use the same conditions for both primers pairs. The mitochondrial primer was chosen so that it could not amplify in the nuclear genome and vice versa.

### rtPCR condition

Real time PCR was performed using a BioRad CFX connect Real-time PCR detection System. The PCR recipe was 2× Perfecta SYBR Green SuperMix for iQ (QuantaBio, Beverly, MA, USA, WHR: 733-1249), 0.2 $\mu$M of each primer, for a final volume of 20 $\mu$l . The PCR temperature cycling used: initial denaturing at 94 °C for 4 min, followed by 45 cycles of denaturing at 94 °C for 30 s, annealing at 60 °C for 30 s and extension at 72 °C for 1 min.

### Experiment

For both the mitochondrial and the nuclear primers, 96 replicas (a whole plate) of the same identical rtPCR were produced. A 2 ml PCR mix was created (as described the section “rtPCR condition”), to which 4 $\mu$l of extracted DNA was added. The mix was spread on a PCR plate adding 20 $\mu$l of it into each well. Serial dilutions for both primers were used to estimate PCR efficiency with the $\Delta C_T$ method. The initial rtPCR mix was serially diluted into rtPCR mix without DNA, by a factor of 5, for six steps. There were 16 replicas for each dilution leading to a total of 16 × 6 = 96 reactions. The use of dilutions reveals changes in PCR efficiency and gives an indication of precision.

The DNA copy numbers were estimated for each tissue based on four different rtPCR reactions: nuclear DNA, mitochondrial DNA, nuclear DNA diluted by 10, and mitochondrial DNA diluted by 10. Each rtPCR reaction was replicated 24 times, giving a total number of 4 × 24 = 96 (a complete 96 well plate) reactions.

### Data analysis

The data analysis algorithm is available in an R package developed specifically for the analysis of rtPCR results. The package, together with the codes used to generate the graphs and tables are included in the Additional file 1. By fitting a robust linear regression line to the base two logarithm of the signal $(\log _2S)$ against the cycle number (*c*), the efficiency (slope of the regression line) and intercept (*I*) associated with each rtPCR reaction is estimated. The fitting proceeds by finding the middle point as the couple ($c_m,\log _2S_m$), where $\log _2S_m$ is closest to middle between the maximal and minimal signal $\frac{\max (\log _2S) + \min (\log _2S)}{2}$. Forcing passage of the fitted line through the middle point ensures that the line fits the exponential phase of the signal.

The relative copy number between two experiments is defined as $\frac{N_A}{N_B}=2^{I_A-I_B}$ which is estimated by taking the difference in the average intercept computed over replicated reactions,

$$\begin{aligned} \text {estimation of } \left(\frac{N_A}{N_B}\right) = 2^{ \left(\overline{I_A}-\overline{I_B}\right)}=2^{\hat{\Delta }I} \end{aligned}$$

The average intercept is assumed to follow a Normal distribution, which is justified by inspection of the results from 96 replicas. The 95% confidence interval for $\Delta I$ can therefore be estimated as:

$$\begin{aligned} C.I.&={\hat{\Delta }I} \pm W; W \\&=q_{0.975}(t_{n_A+n_B -2})\times \sqrt{\frac{Var[I_A]}{n_A}+\frac{Var[I_B]}{n_B}}\,, \end{aligned}$$

where $q_{0.975}$ is the 97.5% quantile of the t distribution, $t_\nu$ is the *t* distribution with $\nu$ degrees of freedom, and $n_A$ and $n_B$ are the number of replicas for A and B respectively and the variances are estimated from the replicated values. The resulting confidence interval for the relative copy number is

$$\begin{aligned} \frac{N_A}{N_B} \times 2^{\pm W}\,, \end{aligned}$$

which shows $2^W$ as a confidence ratio (C.R.). The *C*.*R* tells us that the interval $[\frac{N_A}{N_B}\times \frac{1}{C.R. }, \frac{N_A}{N_B}\times C.R.]$ captures the actual copy number with a probability of $95\%$. The boundaries for the confidence interval of the actual relative concentration, can be calculated by multiplying and dividing the estimated relative concentration by the *C*.*R*. Baseline noise in a rtPCR reaction is estimated by taking the highest point for which the first derivative of the signal as a function of the cycle number is negative. The threshold to calculate the $C_T$ value is chosen by taking the highest value for the baseline in an experiment. When relative concentrations are calculated between two samples, the same threshold is used to calculate the $C_T$ value for both.

## Findings and discussion

### Problem

The phases of an rtPCR reaction are:

Phase I: Lag phase: The signal is too low for the detector, only the noise is visible.
Phase II: Exponential phase: Signal grows exponentially with the number of cycles.
Phase III: Saturation phase: Signal increases sub-exponentially, or not at all as the PCR reaction saturates.

The dynamics of the PCR reaction can only be observed during phase II, during which the signal can be modelled by the exponential function $S=\alpha NE^c$. In this equation, *N* is the number of DNA copies at the start of the experiment, *S* is the signal, $\alpha$ is an unknown constant relating the copy number to the signal intensity and *c* is the cycle number. The constant $\alpha$ is related to parameters such as detection efficiency or fluorescence per base pair. It is assumed that $\alpha$ is constant and does not depend on the sample.

The standard algorithm to analyse rtPCR is the $\Delta C_T$ method. A signal threshold *T* is chosen, a little above the noise level. The $C_T$ value is defined as the cycle number at which the signal crosses the threshold. It is calculated by taking a linear interpolation between the first signal value above the threshold and the one immediately below, then taking $C_T$ as the value at which the line intersects the chosen threshold. If there are two samples, *A* and *B*, for which rtPCR signal has been obtained this yields an equation relating the initial copy numbers of the two samples.

$$\begin{aligned} T=\alpha N_A E_A^{C_T^A}=\alpha N_B E_B^{C_T^B} \text { or }\frac{N_A}{N_B}=\frac{E_B^{C_T^B}}{E_A^{C_T^A}} \end{aligned}$$

Assuming equal efficiency for both reactions, $E_A=E_B=E$, the equation becomes

$$\begin{aligned} \frac{N_A}{N_B}=E^{\Delta C_T}\,, \end{aligned}$$

where $\Delta C_T=C_T^A-C_T^B$ is the difference in the $C_T$ values. The $\Delta C_T$ method has a few clear flaws, which have already been pointed out and demonstrated by Karlen et al. [25]. The first one is the assumption of equal efficiency which is essential to this method. If the fragments used are not the same, as is the case for the quantification of mtDNA, the reaction needs to be optimised to have equal efficiency. If PCR efficiency depends on initial DNA concentration, as some results suggests [24], this would introduce errors in the measurements.

### Proposed solution

The objective of rtPCR is to measure the relative initial copy number $\frac{N_A}{N_B}$ between two samples. Taking the logarithm in the equation used for the $\Delta C_T$ method leads to the equation we fit to the exponentially increasing signal,

$$\begin{aligned} \log _2 S=\log _2(\alpha N)+ c\log _2E, \quad \text { where }\log _2(\alpha N)=I. \end{aligned}$$

The slope $\log _2E$ is related to the efficiency and $I=\log _2(\alpha N)$ is the intercept or the value of the signal extrapolated to the start of the reaction at $c=0$. We propose to estimate the values of the intercept and the slope by fitting a regression line to several consecutive pairs (c, S) chosen from the exponential phase of the reaction. If we have intercepts for two samples A and B we obtain

$$\begin{aligned} \frac{N_A}{N_B}=2^{I_A-I_B}\, \end{aligned}$$

which requires only a constant value of $\alpha$, but gives correct results even when the efficiencies for A and B are different. The slope of the regression gives an estimate of the efficiency for a single reaction without having to perform dilutions. Accuracy can be increased by replicating the reactions several times. Thus, it is possible to compare samples with different efficiencies, which reduces the difficulties in optimising the PCR reactions and improves precision.

In our analyses, we used the robust line fitter that minimises the median of the squared residuals, whereas the least squares estimator minimises the mean of the squared residuals. The line passing through the mid-point has equation $\log _2S_m + (c-c_m) \log _2E$ and to determine the value of $\log _2(E)$, we fix it such that the median over all measured couples (*c*, *S*) of $(\log _2S -\log _2S_m - (c-c_m) \log _2E)$ is smallest. Taking the median means that the line can tolerate up to one half of the measured couples not to be near the regression line, which is the case for the phases I and III. The minimisation has to be done numerically and the package supplied in the Additional file 1 will perform the necessary computations.

### Results

**Fig. 1:**
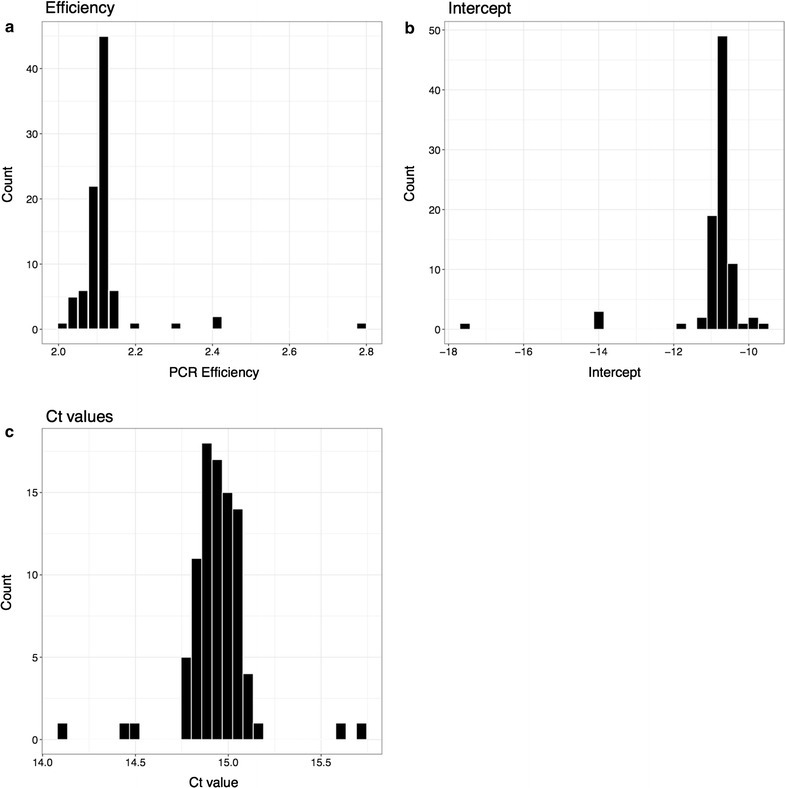
Histograms showing the distribution of efficiency, intercept and $C_T$. Data shown from 94 replicas. For each reaction, the robust regression method is used to compute the efficiency and intercept

Figure 1 shows the results of repeating the same reaction 96 times. The efficiency and intercept, calculated using the robust regression, as well as the $C_T$ values are shown. In all three cases, the values group together with five outliers. These outliers are not PCR failures. They represent genuine variation in PCR performance on an identical rtPCR mix. These results justify the use of a normal approximation.

**Fig. 2:**
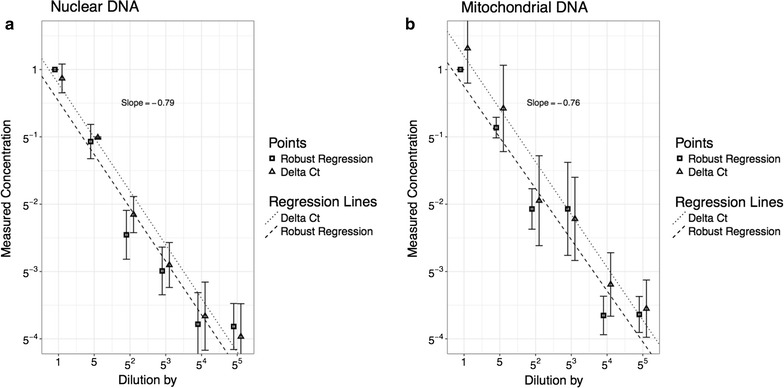
Dilution series: DNA concentration in the PCR mix is serialy diluted by a factor 5, six times. The DNA concentration is measured using the robust regression method and the $\Delta C_T$ method. Each concentration is prepared in 16 replicas. The same procedure is reproduced for both nuclear and mitochondrial DNA. Regression lines are fitted using the least squared method. The data has been shifted slightly left and right of the true value for illustration purposes

Figure 2 shows the result of the dilution series in which the relative concentrations relative to the initial sample are known. For both nuclear and mitochondrial DNA, the relative concentrations were estimated with the $\Delta C_T$ algorithm as well as the new robust regression method. The vertical axis is the logarithm to base five of the relative concentration and since we dilute by a factor of five, the points should lie on a line with slope $-1$.

It can be seen that the robust regression gives, in both cases, a slightly better slope than the $C_T$ method. This difference does not appear to be significant. The dilution series can be used to estimate the efficiency of the PCR using the $C_T$ method. If the efficiency is assume identical in all samples then:

$$\begin{aligned} \frac{N_A}{N_B}=E^{\Delta C_T}\Rightarrow \log _5\frac{N_A}{N_B}=\Delta C_T \log _5 E \end{aligned}$$

**Fig. 3:**
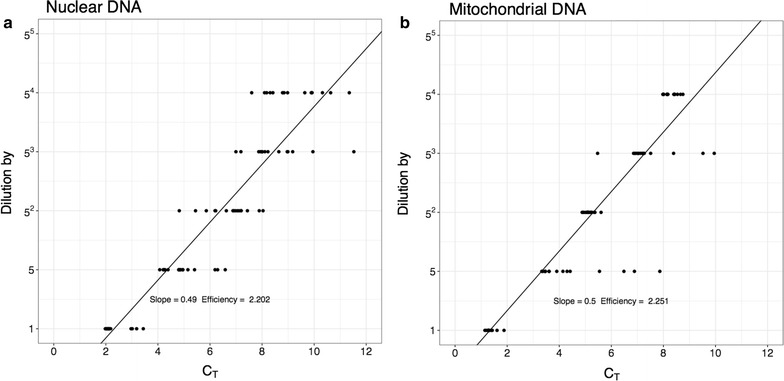
Calculating the efficiency using the $\Delta C_T$ method. The measured $C_T$ values for each dilution are plotted. The slope of the fitted line can be used to estimate the efficiency

The logarithm of the dilution factor, is linearly related to the $\Delta C_T$, and the slope is the log of the efficiency. Results are shown in Fig. 3.

**Table 2:** Relative concentration of samples for each tissue type

Tissue	Sample type	Efficiency	Concentration	Relative error
Breast tumor				
	mtDNA	2.08	129.3	1.29
	mtDNA/10	2.13	6.87	1.47
	Nuclear	2.02	1	1
	Nuclear/10	1.99	0.16	1.4
Breast normal				
	mtDNA	2.19	132.13	1.4
	mtDNA/10	2.02	38.54	1.36
	Nuclear	2.03	1	1
	Nuclear/10	2.02	0.09	1.38
Colon tumor				
	mtDNA	2.13	132.72	1.35
	mtDNA/10	2.1	12.95	1.32
	Nuclear	2.01	1	1
	Nuclear/10	2.01	0.09	1.45
Colon normal				
	mtDNA	2.11	219.19	1.5
	mtDNA/10	2.11	20.82	1.56
	Nuclear	2.05	1	1
	Nuclear/10	1.99	0.2	1.51
Prostate tumor				
	mtDNA	2.17	134.14	1.41
	mtDNA/10	2.11	15.79	1.39
	Nuclear	2.04	1	1
	Nuclear/10	2	0.16	1.47
Prostate normal				
	mtDNA	2.12	172.65	1.49
	mtDNA/10	2.08	22.21	1.47
	Nuclear	2.02	1	1
	Nuclear/10	2.01	0.13	1.55

The nuclear DNA concentration is taken as reference, and is therefore 1. The relative error (confidence ratio) is also 1 as there is no uncertainty associated with it. The other concentrations are relative to nuclear DNA and the relative error associated with it. The values for the efficiency are the average taken over the replicas

**Table 3:** Concentrations calculated using the $\Delta C_T$ method in prostate tumor sample

	Concentration	Relative error
mtDNA	288.33	1.52
mtDNA/10	23.45	1.44
Nuclear	1.00	1.00
Nuclear/10	0.11	1.52

The confidence ratio is estimated using the t-interval

Table 2 shows the estimates of mtDNA copy numbers based on the relative concentration of mitochondrial DNA compared to nuclear DNA. The numbers range from 100 to 150 for all samples, which represent half the total number of mtDNA copies per cell. The confidence ratios are around 1.3. The ratio between the measured concentrations of mtDNA and diluted mtDNA should be 10. Taking into account the confidence ratios associated with the measurement, the diluted samples have indeed a copy number 10 times below their un-diluted counter-parts. Observing a C.R. of 1.3 for a mitochondrial copy number of 200, corresponds to having a 95% confidence interval between 150 and 260. This precision was achieved with 24 replicas. The C.R. decays very slowly as a function of the number of samples. Using a robust regression to analyse rtPCR data presents major advantages over the $\Delta C_T$ method. First, it does not make the assumption of identical PCR efficiency between two samples. This reduces potential biases and allows for the comparison of fragments/samples with clearly different efficiencies. It also allows the estimation of PCR efficiency without performing dilution series. If the efficiency depends on the initial copy number, it would be an additional source of bias for the $\Delta C_T$ algorithm. Figure 3 shows efficiency calculations for the dilutions series. For comparison, the prostate tumor tissue is analysed using the $\Delta C_T$ method (shown in Table 3). The results are higher than those estimated using the robust regression. They are, however, coherent if the larger confidence ratio is taken into account. Karlen et al. [25] also shows that the $\Delta C_T$ method performs well in the case of identical efficiencies, but may be a bad choice in other circumstances. The robust regression method offers an alternative way to analyse rtPCR data which has important advantages.

## Limitations

The analysis method proposed here is limited to the analysis of rtPCR results. It can be used with any standard rtPCR output data and represents an improvement from the $\Delta C_T$ method. However, a large number of replicas is still needed to achieve low C.R.

## Additional file

**Additional file 1.** rtPCR package for R. The R package containing the software tools to perform robust regression analysis of rtPCR data. The package is in binary format and can be installed into R.

## Abbreviations

rtPCR: real time polymerase chain reaction
DNA: deoxyribonucleic acid
mtDNA: mitochondrial DNA
tRNA: transcription ribonucleic acid
